# Knockdown H19 Accelerated iPSCs Reprogramming through Epigenetic Modifications and Mesenchymal-to-Epithelial Transition

**DOI:** 10.3390/biom14050509

**Published:** 2024-04-23

**Authors:** Ruizhen Sun, Ximei Zhang, Tiantian Gong, Yue Zhang, Qi Wang, Chenyao He, Jielan Ju, Chunmiao Jin, Wenxin Ding, Jingnan Gao, Jingling Shen, Qiuming Li, Zhiyan Shan

**Affiliations:** 1 Department of Histology and Embryology, Harbin Medical University, Harbin 150081, China; srz_1983@hrbmu.edu.cn (R.S.); zhangxm@hrbmu.edu.cn (X.Z.); gtt915@126.com (T.G.); zhangyue613@126.com (Y.Z.); krystal_wangqi@163.com (Q.W.); hecy502337626@126.com (C.H.); m17860506681@163.com (J.J.); j18845181359@126.com (C.J.); shiqi630@yeah.net (W.D.); gjingnan@126.com (J.G.); 2 Institute of Life Sciences, College of Life and Environmental Science, Wenzhou University, Wenzhou 325035, China; jingling_shen@wzu.edu.cn

**Keywords:** H19, cell reprogramming, mesenchymal-to-epithelial transition

## Abstract

H19 is an essential imprinted gene that is expressed to govern normal embryonic development. During reprogramming, the parental pronuclei have asymmetric reprogramming capacities and the critical reprogramming factors predominantly reside in the male pronucleus. After inhibiting the expression of H19 and Gtl2, androgenetic haploid ESCs (AG-haESCs) can efficiently and stably support the generation of healthy SC pups at a rate of ~20%, and double-knockout parthenogenetic haESCs can also produce efficiently. Induced pluripotent stem (iPS) cell reprogramming is thought to have a characteristic epigenetic pattern that is the reverse of its developmental potential; however, it is unclear how H19 participates in iPS cell reprogramming. Here, we showed that the expression of H19 was transiently increased during iPSC reprogramming. H19 knockdown resulted in greater reprogramming efficiency. The genes associated with pluripotency showed enhanced expression during the early reprogramming process, and the Oct4 promoter was demethylated by bisulfite genomic sequencing analysis. Moreover, expression analysis revealed that the mesenchymal master regulators associated with epithelial-to-mesenchymal transition (EMT) were downregulated during reprogramming in H19 knockdown. These findings provide functional insight into the role of H19 as a barrier to the early reprogramming process.

## 1. Introduction

Induced pluripotent stem cells (iPSCs) are generated from somatic cells via the over-expression of transcription factors, such as Oct4, Sox2, Klf4, and c-Myc, or small-molecule compounds [1,2,3,4]. They provide a way to produce patient-specific pluripotent stem cells with fewer ethical issues in order to facilitate the study and possible treatment of degenerative diseases. However, the low efficiency of generating iPS cells is still a barrier to iPS reprogramming. Genomic imprinting is an epigenetic phenomenon that plays an important role in development and diseases [5,6,7,8,9,10,11,12]. Previous studies have reported that aberrant epigenetic silencing of some imprinted gene clusters, such as Dlk1-Dio3, tended to hinder mouse developmental potency, and treatment with the methyltransferase inhibitor could attenuate hypermethylation of the Dlk1-Dio3 locus and enable high-grade chimerism in mice [13]. Located in the mouse chromosome 7 H19/Igf2 loci, H19 encodes important imprinted genes. The transcription of H19 gives rise to the non-coding mRNA that is a precursor of several microRNAs (miR-675) that govern normal embryonic development; its expression is down-regulated after birth [14]. Somatic cell nuclear transplantation (SCNT) and induced pluripotent stem cell (iPSC) technologies can be employed to change cell fates via reprogramming. SCNT studies indicated that parental pronuclei have asymmetric reprogramming capacities and that the critical reprogramming factors predominantly reside in the male pronucleus [15]. The injection of androgenetic haploid embryonic stem cells (AG-haESCs) with H19 and Gtl2 defects into oocytes supports the generation of healthy semi-cloned (SC) pups at a rate of ~20% [16]. Moreover, the use of parthenogenetic haploid ESCs (PG-haESCs) via the removal of H19 and Gtl2 DMRs exhibited highly similar gene expression profiles to those when using AG-haESCs; double-knockout PG-haESCs can efficiently produce SC pups [16]. In addition, embryonic germ cell extracts improved the efficiency of iPS cells by erasing the imprint, such as in H19 and Gtl2 [17]. Inhibited H19 reduced the proliferation of human embryonic carcinoma cells (hECs) and embryonic stem (hES) cells [18]. However, in H19, there were clone-to-clone variations and no consistent differences between ES cells and iPSCs [19]. The low cloning efficiency of SCNT and the differences between iPSCs and embryonic stem cells (ESCs) may be caused by incomplete or aberrant reprogramming, which reaffirms the importance of cell fate plasticity. Since these aspects are significant barriers, we detect how H19 imprinting is reprogrammed during iPS cell derivation, which may lead to the eventual production of therapeutically suitable iPS cells.

## 2. Materials and Methods

### 2.1. Experimental Animals

C57BL/6J, DBA/2J, and NOD-SCID mice (6–8 weeks old) were purchased from Vital River (Beijing, China). All mice were housed at 23 ± 3 °C at 55 ± 15% humidity with 12 h light/dark cycles and were sacrificed by neck dislocation to collect samples. This study was carried out in strict accordance with the recommendations in the Guide for the Care and Use of Laboratory Animals and was approved by the Institutional Animal Care and Use Committee at Harbin Medical University (the approval code is HMUIRB20190012).

### 2.2. Construction of H19-shRNA Expression Vector and Lentiviral Infection

H19-shRNA (shH19) was cloned into the pSIH1-H1-GFP shRNA Vector (System Biosciences, Palo Alto, CA, USA) according to the manufacturer’s protocol; the vector information is shown in Figure S1. The 293T cells were transfected with the sh-H19 expression vector using a lentivirus package plasmid mix. The medium containing lentiviral particles was harvested after 48 h transfection and used to transduce MEF cells. The 293T cells were maintained in high-DMEM (BI, Migdal HaEmek, Israel) culture medium containing 10% FBS (BI, Israel). 

### 2.3. Generation of iPS Cells

Mouse embryonic fibroblasts (MEFs) were isolated from E13.5 B6D2F1 mouse embryos and cultured in high-DMEM with 10% FBS. Then, 1 × 10^5^ MEFs were plated on 6-well plates and transfected with retroviruses of pMXs-Oct4, pMXs-Sox2, pMXs-Klf4, and pMXs-c-Myc MEFs after 24 h. On the next day, MEFs were transfected again in MEF medium containing 10% FBS and labeled as day 0 (D0). MEFs containing OSKM transgenes were transduced with shNC or shH19 lentiviral particles at D0. On D3, the infected MEFs were reseeded on feeder layers, and the medium was substituted for the iPS cell medium. The iPS clones were manually picked and maintained in KO-DMEM with 20% FBS, 1× GlutaMax (Invitrogen, Waltham, MA, USA) and P/S, 1000 unit/mL recombinant mouse LIF (Chemicon(millipore), Billerica, MA, USA), and 100μM 2-mercaptoethanol (Invitrogen, Waltham, MA, USA). 

### 2.4. RT-PCR and RT-qPCR Expression Analysis

The RNA was extracted using TRIzol Reagent (Invitrogen, Waltham, MA, USA). cDNA was synthesized using a High-Capacity cDNA Reverse Transcription kit (Transgen Biotech, Beijing, China). Primers used to measure endogenous expression are summarized in Table S1. RT-PCRs were performed using a Taq polymerase kit (TransGen Biotech, Beijing, China). Amplification products were confirmed in 2% agarose gel containing GelStain (TransGen Biotech, Beijing, China) for visualization with AlphaImager^®^ HP (Alpha Innotech, San Leandro, CA, USA). RT-qPCRs were performed using TransStartTM Top Green qPCR Super Mix (TransGen Biotech, Beijing, China). Gene expression was detected using the BioRad real-time PCR system. The housekeeping gene Gapdh was employed as a control for analyzing relative mRNA levels. The 2-ΔΔCt method was used to analyze gene expression data. Each sample was examined in triplicate. 

### 2.5. Alkaline Phosphatase Staining and Cell Proliferation Assay

Alkaline phosphatase activity was detected using the alkaline phosphatase substrate kit (Beyotime, Beijing, China). A cell proliferation assay was performed using a Cell Counting Kit (CCK8, BestBio, Shanghai, China) according to the manufacturer’s guidelines.

### 2.6. Flow Cytometric Assay

Cells were dissociated with trypsin for 5 min, neutralized using a medium, and gently centrifuged (1000× *g*, 10 min). The supernatants were removed and the cells were resuspended in DPBS with 2% normal goat serum at RT for 30 min. Then, the cells were cultured with SSEA1 (1:100, Santa Cruz, Dallas, TX, USA) solution for 1 h at RT, washed 3 times (1000× *g*, 5 min) with DPBS, and incubated with the secondary antibody (donkey anti-rabbit IgG-488, 1:500, Invitrogen, Waltham, MA, USA) for 20 min at RT in the dark. Afterward, cells were washed 3 times (1000× *g*, 5 min) and resuspended in DPBS in a Flow cytometer (Cytomics FC 500, Beckman Coulter, Brea, CA, USA). The data were analyzed using FlowJo flow cytometry software (version 7.6.1, Tree Star Inc., Ashland, OR, USA).

### 2.7. Bisulfite Genomic Sequencing

Genome DNA of cells was extracted using EasyPure™ Genomic DNA Kit (Transgen Biotech, Beijing, China). A Bisulfite reaction was carried out using an EZ DNA Methylation Kit (Zymo Research, Los Angeles, CA, USA) according to the manufacturer’s recommendations. For the PCR, the samples were amplified using Taq™ Hot Start (TaKaRa, Osaka, Japan), and the products were recovered using the EasyPure Quick Gel Extraction Kit (Transgen Biotech, Beijing, China). Next, the products were cloned into pEASY-T3 (Transgen Biotech, Beijing, China) and translated into Trans1-T1. Then, ten clones were randomly selected and sequenced by a company (Invitrogen, Waltham, MA, USA). Allele sizes were approximated on the basis of the known sizes of various inbred strains.

### 2.8. In Vivo and In Vitro Differentiation 

In vitro differentiation showed the formation of embryoid bodies (EBs). iPSCs were treated with single-cell suspension and plated in low-adherence dishes in iPSCs medium without LIF. Then, the medium was refreshed every other day and the plated EBs were harvested to extract total RNA on day 3 or 6 for RT-PCR. In vivo differentiation was detected via teratoma formation. Then, 1 × 10^6^ iPSCs were injected subcutaneously into NOD–SCID mice. Next, the mice were sacrificed 4–6 weeks after cell injection; teratomas were fixed in 4% paraformaldehyde, processed for paraffin sectioning, and stained with hematoxylin and eosin (HE) for histological examination. 

### 2.9. Statistical Analysis

At least three clones were used to establish iPSCs. At least three iPSCs were used to perform the experiment at least 3 times Statistical data were analyzed via *t*-test, with significant differences of * *p* < 0.05, ** *p* < 0.01, and *** *p* < 0.001. 

## 3. Results

### 3.1. H19 Knockdown Contributes to iPSCs Reprogramming

**Figure 1:**
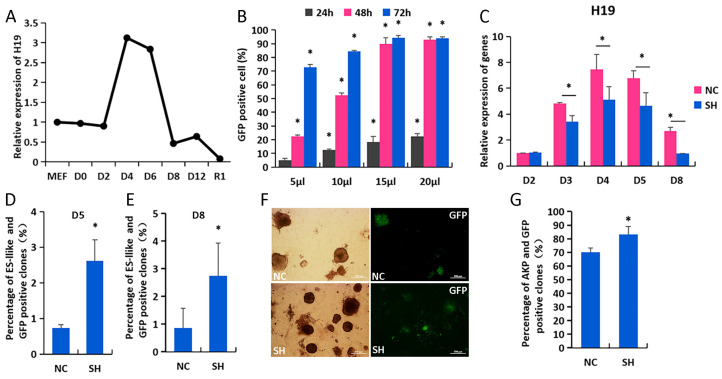
H19 knockdown contributes to iPSC reprogramming. (**A**) Expression of H19 in iPSC reprogramming; (**B**) GFP-positive cell rate after virus infection with different concentrations; (**C**) interference efficiency of H19 was detected by RT-qPCR in NC (shNC) and SH (shH19) groups; (**D**,**E**) percentage of GFP and ES-like positive clones on D5 and D8, respectively; (**F**,**G**) results of AKP staining for NC (shNC) and SH (shH19) groups, and statistical analysis of AKP and GFP positive ES-like clones in NC and SH groups. * *p* < 0.05, scale bars = 500 μm.

H19 localizes to the imprinted H19-Igf2 gene cluster on mouse chromosome 7 and is essential for imprinting gene expression in mammals. H19 was strongly repressed in iPSCs compared to MEFs; meanwhile, transient increased H19 was observed in induced MEFs from D2 to D8 during OSKM reprogramming (Figure 1A). This may suggest that the high expression level of H19 had a negative impact on reprogramming. We designed three shRNAs directed against H19 and infected MEF cells with lentiviral particles encoding shRNA sequences (Table S1). It was found that 15 µL H19-shRNA-1 (shH19) resulted in the greatest knockdown of H19 mRNA, as evaluated by RT-qPCR and GFP-positive cells with immunofluorescence (Figure 1B,C and Figure S1B,C). These results were used for subsequent studies. Equal numbers of MEFs were OSKM infected with lentiviral particles encoding for shRNA or shH19 control. Fluorescence microscopy was used to visualize the reprogramming of shNC and shH19 MEFs (Figure S2). After 5 days, shH19 MEFs contained increased levels of GFP-positive ES-like clones compared to shNC MEFs (Figure 1D,E, D5, shH19 2.5% and shNC 0.8%; D8, shH19 3.5% and shNC 0.9%); moreover, the quantification of changes demonstrated an increased AP staining intensity (Figure 1F,G). This indicates that H19 knockdown results in greater reprogramming efficiency, and suggests that H19 is a barrier to reprogramming.

### 3.2. H19 Knockdown Facilitates Expression of Pluripotent Genes during iPSCs Reprogramming

**Figure 2:**
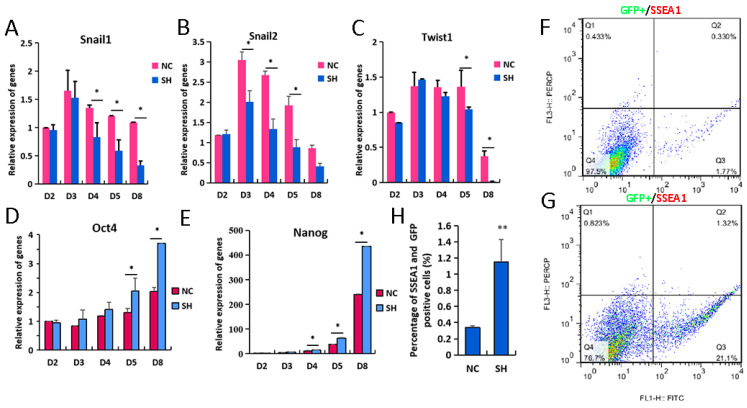
H19 knockdown facilitates the expression of pluripotent genes during iPSCs reprogramming. (**A**–**C**) The expressions of Snail1, Snail2, and Twist1 were analyzed via real-time PCR during iPSC reprogramming, respectively; (**D**,**E**) the expressions of Oct4 and Nanog during iPSCs reprogramming using real-time PCR analysis, respectively; (**F**,**G**) the expressions of SSEA1 and GFP in NC and SH groups by FCAS, respectively; (**H**) statistical analysis of GFP- and SSEA1-positive cells in NC (shNC) and SH (shH19) groups for (**F**,**G**) results. * *p* < 0.05, ** *p* < 0.01.

To compare the molecular roadmaps of H19 in OSKM reprogramming, we analyzed the gene expression relevant to established reprogramming stages. MET occurs on D3–D5 for OSKM reprogramming, as evident from the downregulation of fibroblast-specific genes (Snail1, Snail2, Twist1) by RT-qPCR detection (Figure 2A–C). Snail2 starts as early as D3 in OSKM expression, showing more advanced kinetics. Marked by the upregulation of pluripotency-specific genes (Oct4, Nanog), the maturation stage of reprogramming is largely advanced for shH19 reprogramming (Figure 2D,E). Compared with the activation of Nanog, endogenous Oct4 is activated in a secondary activation wave during both shNC and shH19 reprogramming; however, the upregulation of Nanog was more advanced in shH19 versus shNC reprogramming. In addition, FACS data showed that the rate of GFP+SSEA1+ cells was increased in shH19-MEFs versus shNC-MEFs on D9 (Figure 2F–H), which is in accordance with our RT-qPCR data, showing an increasing expression of pluripotency-specific genes during shH19 reprogramming.

### 3.3. Dynamic Loss of H19 Leads to Diverse Reprogramming Efficiency

**Figure 3:**
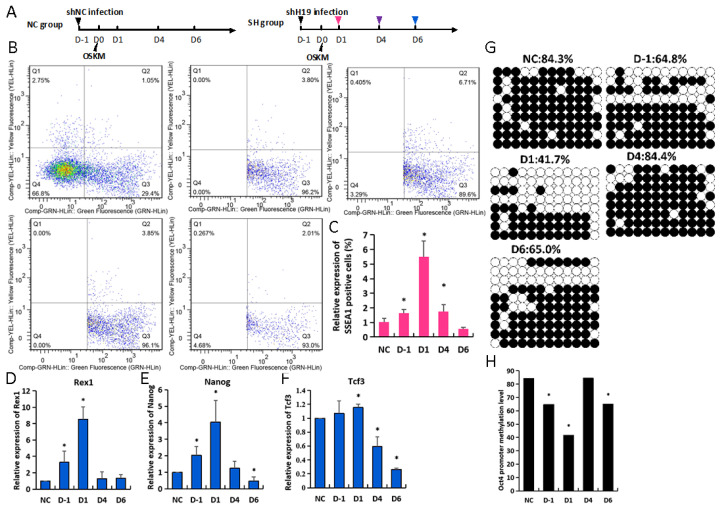
Dynamic loss of H19 leads to diverse reprogramming efficiencies. (**A**) A schematic of H19 interference during iPSC reprogramming; the various colored triangles represent shH19 virus transfection points. (**B**,**C**) The expression of SSEA1 and GFP was detected in NC, D-1, D1, D4, and D6 groups via FACS analysis and statistical analysis of GFP- and SSEA1-positive cells; (**D**–**F**) the expression of Rex1, Nanog, and Tcf3 was detected by RT-qPCR; (**G**,**H**) methylation analysis of Oct4 promoter H19 knockdown on iPSC reprogramming in NC, D-1, D1, D4, and D6 groups using bisulphite sequencing. Open and filled circles indicate unmethylated and methylated CpG dinucleotides, respectively. * *p* < 0.05.

Because the peak abundance of H19 expression was from D4 to D6, we postulated that the reprogramming may be pushed into a different efficiency when H19 was inhibited in different reprogrammed periods in MEFs. MEFs were treated with the shH19 regimen 1 day before OSKM (D-1), 1 day after OSKM (D1), or 4 or 6 days after OSKM (D4 or D6) (Figure 3A, Figures S3 and S4). FACS against SSEA1 in group D1 displayed a significant increase compared with shNC under all tested conditions (Figure 3B,C). The pluripotent gene (Rex1, Nanog, Tcf3) levels were significantly increased in groups D-1 and D1; the D1 group was the highest (Figure 3D–F). Because the H19 loss induced at different times leads to diverse reprogramming efficiencies, we evaluated the mechanistic role of H19 in accelerated reprogramming regulation. Most of the cells exhibited DNA methylation loss in the promoter regions of Oct4 under all tested groups compared with the shNC group (Figure 3G,H). The methylated levels of the Oct4 promoter were significantly decreased in group D1(41.7%); they showed a slight decrease in the other H19 group (D-1, 64.8%; D6, 65%). Collectively, these data indicate that H19 knockdown improved reprogramming under all the conditions tested. The reprogramming improvement was most prominent in MEFs that were treated with the shH19 regimen 1 day after OSKM, which led to an accelerated methylation loss in the Oct4 promoter.

### 3.4. H19-Depleted iPSCs Exhibit Reduced Self-Renewal

**Figure 4:**
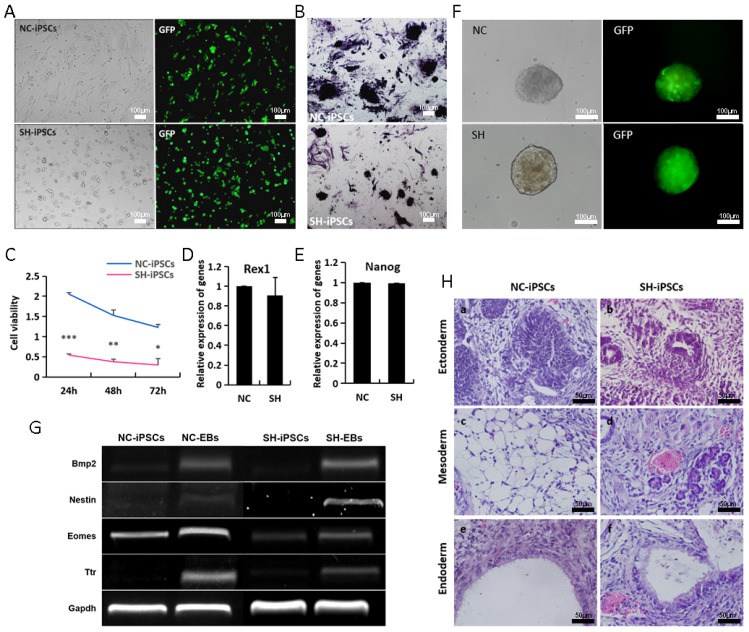
H19-depleted iPSCs exhibit reduced self-renewal. (**A**,**B**) Morphology and AKP staining of NC-iPSC and SH-iPSC lines; (**C**) proliferating capability analysis of NC-iPSCs and SH-iPSCs using CCK8 kit; (**D**,**E**) the expressions of pluripotent genes Rex1 and Nanog in NC-iPSC and SH-iPSC lines by RT-qPCR analysis, respectively; (**F**,**G**) morphology and germ layer genes of EB from in vitro differentiation of NC-iPSC and SH-iPSC lines, respectively; (**H**) teratomas containing multiple tissues analyzed histologically with H&E staining: (**a**,**b**): neural tube, (**c**): adipose tissue, (**d**): blood vessel, (**e**,**f**): respiratory tract. * *p* < 0.05, ** *p* < 0.01 and *** *p* < 0.001. Original images of (**G**) can be found in Supplementary Materials.

To further characterize established iPSCs lacking H19, we randomly picked the GFP-positive colonies in both group D1 (SH-iPSCs) and shNC (NC-iPSCs). We synchronously passaged these in iPSC medium for use in subsequent studies. The morphological results did not reveal differences between NC-iPSCs, SH-iPSCs, and ESCs; however, SH-iPSCs showed a reduced colony size compared with NC-iPSCs (Figure 4A). Similarly, the AP staining levels decreased (Figure 4B). CCK8 analysis showed decreased SH-iPSC numbers compared to NC-iPSCs after 48 h or 72 h in the culture, indicating that SH-iPSCs saw reduced colony formation (Figure 4C). The results of expression showed that self-renewal genes, such as Nanog, Rex1 and Oct4, was not changed in SH-iPSCs relative to NC-iPSCs (Figure 4D,E and Figure S5). EB formation represents a stringent in vitro model for evaluating ES cell differentiation. To further understand the expression of differentially expressed genes, we used EB formation as a tool to functionally evaluate the role of H19 during ES cell differentiation. SH-EBs were a similar size to NC-EBs (Figure 4F). The data showed that differentiation genes, including Nestin (ectoderm), Bmp2 (mesoderm), Ttr, and Eomes (endoderm), were not changed between SH-iPSCs and NC-iPSCs (Figure 4G and Figure S6). Teratoma formation was subsequently used to evaluate the potential of SH-iPSCs to differentiate in vivo into cells represented in the three germ layers. SH19-iPSCs and NC-iPSCs were injected subcutaneously into SCID-beige mice that were subsequently dissected after 4 weeks of development. H&E histological analysis showed both SH-iPSC- and NC-iPSC-derived teratomas contained cells with three germ layers (Figure 4H). No differences were observed in these two kinds of iPSC demonstrating that H19 knockdown is not important for ES cell differentiation.

## 4. Discussion

Imprinted genes are very important in the development of mammalian embryos, and their abnormal expression leads to abnormal embryonic development and even tumors [20,21,22,23]. H19 encodes an important imprinted gene located in the mouse chromosome 7 H19/Igf2 loci [14,24]. The function of imprinted H19 long non-coding RNA remains controversial. Recently, Luo et al. reported on the offspring production of ovarian organoids derived from spermatogonial stem cells via the transduction of H19 imprint genes, transcript factors Stella and Zfp57, and the inactivation of Plzf with chromatin reorganization [25]. Previous studies indicated that the H19 imprint gene was essential in the SCNT of reprogramming [16], but it is not clear in iPS reprogramming. In the present study, our data demonstrated that H19 in ESCs was significantly less expressed than in MEFs, but it saw transient hyper-expression during iPSC reprogramming. Meanwhile, we clearly indicated that the suppressed H19 during iPS reprogramming improved the efficiency of ES-like clones and the expression of the pluripotency genes, Oct4, Nanog, and Rex1, and the surface antigen, Ssea1. In a previous report, AG-haESCs were shown to efficiently and stably support the generation of healthy SC pups at high rates by inhibiting H19 and Gtl2; via the removal of H19 and Gtl2 DMRs, PG-haESCs exhibit highly similar gene expression profiles to those of AG-haESCs and can efficiently produce SC pups by injection oocytes [16]. Therefore, these data suggested that H19 knockdown was conducive to improving both SCNT and iPSC reprogramming. Oct4 has been shown to be a key transcript factor in the reprogramming process. Activated Oct4 promoters (OCT4 promoters of DNA demethylation) enhanced iPSC formation, and along with the quartet factors, exhibited typical ESC morphology, gene expression patterns and developmental potential [26]. Our results revealed that H19 expression was dynamic during iPS reprogramming, and a high expression appeared in D3-D6. Then, at different times, H19 inhibition demonstrated that the pluripotent gene markers, Ssea1, Rex1, Nanog and Oct4, saw the highest H19 knockdown expressions in the D1 group. Furthermore, the Oct4 methylation promoter was lowest in the D1 group. This was inconsistent with previous reports on human stem cells where H19 knockdown resulted in a decrease in the expression of the pluripotency markers, Oct4, Nanog, TRA-1-60, and TRA-1-81, and in the up-regulation of SSEA1 and E-Cadherin [18]. This might be due to the differences between species; H19’s role and mechanism in reprogramming should be tracked in different species. However, the proliferation ability of H19 knockdown iPSCs was consistent with Zeira’s results regarding inhibited H19 reducing the proliferation of human embryonic carcinoma cells (hECs) and embryonic stem (hES) cells [18]. Furthermore, our data showed that H19 knockdown iPSCs slowed down in vivo differentiation. However, it had no impact on tri-lineage differentiation by EB and teratoma formation. Above all, it provided that the best reprogramming efficiency was achieved with inhibited H19 in D1 during mouse iPSC induction. Bulk RNA sequencing will subsequently reveal the wide-scale changes in reprogramming timing and efficiency upon perturbation of H19, and the regulating mechanism of H19, on Oct4, needs to be further studied.

MET is an essential early step in reprogramming, as it remodels the mesenchymal cytoskeleton and cell surface protein expression to adopt an epithelial-like morphology and facilitates metabolic and cell fate changes [27,28]. The generation of iPSCs from fibroblasts requires a MET orchestrated by suppressing pro-EMT signals [27,28,29]. In some cancers, H19 seems to induce epithelial-to-mesenchymal transition (EMT), with a decreased expression of epithelial markers and an increased expression of mesenchymal markers [30,31,32,33]. In addition, H19 accelerated the invasion of trophoblast cells [9,11]. Our findings demonstrated that inhibited H19 significantly decreased the expression of the EMT-related genes, Snail1, Snail2, and Twist. This role was consistent with previous reports that showed that H19 improved MET in tumors and trophoblasts. Thus, the results above indirectly indicate that suppressed H19 could regulate iPSC reprogramming through MET. In addition, our data showed the Oct4 promoter caused hypomethylation in the H19 knockdown reprogramming, while epigenetic modifications require further elucidation.

In conclusion, we demonstrated that the H19 of imprinted genes had an effect on mouse reprogramming, in that H19 knockdown promoted reprogramming by inhibiting EMT without affecting the pluripotency and in vivo and in vitro differentiation. Nevertheless, H19 knockdown could decrease the proliferation capability. Therefore, further studies are needed to analyze the epigenetic modification or related mechanism of the H19 knockdown in iPSC lines. Such studies are necessary in order to establish more secure and efficient iPSC lines.

## Data Availability

The data presented in this study are available on request from the corresponding author.

## Supplementary Materials

The following supporting information can be downloaded at https://www.mdpi.com/article/10.3390/biom14050509/s1, Table S1. siRNA sequence of H19; Table S2. RT-qPCR primer; Table S3 RT-PCR primer; Figure S1. shH19 virus transfection efficiency; Figure S2. The morphological changes during ips reprogramming; Figure S3. The morphology of GFP-positive cells during shH19 virus infecting MEFs at different times; Figure S4. The morphology of ES clones on D6 and D12 in different groups; Figure S5. shH19 iPSCs exhibit the expression of pluripotent genes, OCT4 and NANOG; Figure S6. EB of SH-iPSC lines-obtained germ-layer genes, NESTIN, BRACHUYURY and AFP.
