# Supplementary material for: Knockdown H19 Accelerated iPSCs Reprogramming through Epigenetic Modifications and Mesenchymal-to-Epithelial Transition
Source: Biomolecules. 2024 Apr 23;14(5):509. doi: 10.3390/biom14050509 (PMC11118134; doi:10.3390/biom14050509)
Supplement: Supplementary file 1 [file biomolecules-14-00509-s001.zip › Tables.pdf]

Supplement Table S1. siRNA sequence of H19

| ShH19 siRNA | primer (5' to 3') |
|---|---|
| siRNA1 | GCTCCACTGACCTTCTAAA |
| siRNA5 | GGTTCAAGAGTGGCTCTGG |
| siRNA8 | CAGTGGACTTGGTACACTG |
| siRNA-NC | GAAGCCAGATCCAGCTTCC |

Supplemental Table S2. RT-qPCR primer

| Gene | Forward primer (5' to 3') | Reverse primer (5' to 3') |
|---|---|---|
| H19 | GAACAGAAGCATTCTAGGCTGG | TTCTAAGTGAATTACGGTGGGTG |
| Snail1 | CACACGCTGCCTTGTGTCT | GGTCAGCAAAAGCACGGTT |
| Snail2 | TGGTCAAGAAACATTTCAACGCC | GGTGAGGATCTCTGGTTTTGGTA |
| Twist1 | GGACAAGCTGAGCAAGATTCA | CGGAGAAGGCGTAGCTGAG |
| Oct4 | AGAGGATCACCTTGGGGTACA | CGAAGCGACAGATGGTGGTC |
| Nanog | GAACGCCTCATCAATGCCTGCA | GAATCAGGGCTGCCTTGAAGAG |
| Rex1 | AAGCTGCCAGCCAGTAACC | CCTTGCGTTCCACCAACTTTC |
| Tcf3 | GACAGAAGTGGAATTTGTGTCCG | AGTGCCTGGTACTTTCTACGAT |
| Gapdh | AGGTCGGTGTGAACGGATTTG | TGTAGACCATGTAGTTGAGGTCA |

Supplemental Table S3. RT-PCR primer

| Gene | Forward primer (5' to 3') | Reverse primer (5' to 3') |
|---|---|---|
| Bmp2 | TGCTTCTTAGACGGACTGCG | CTCCACGGCTTCTTCGTGAT |
| Nestin | GCCACTCCCTTCTCTAGTGC | CTCGCAGAGCCTCTAACTCG |
| Eomes | AAATTCCACCGGCACCAAAC | AAGCCGTGTACATGGAATCGT |
| Ttr | TCCTGTGTCTGACGAGGAATC | CGGGGAAATGCCAAGTGTCT |
| Gapdh | TGAGCCTCCTCCAATTCAACC | CAGAAGGGGCGGAGATGATG |
