# Supplementary figures and images for: Knockdown H19 Accelerated iPSCs Reprogramming through Epigenetic Modifications and Mesenchymal-to-Epithelial Transition
Source: Biomolecules. 2024 Apr 23;14(5):509. doi: 10.3390/biom14050509 (PMC11118134; doi:10.3390/biom14050509)

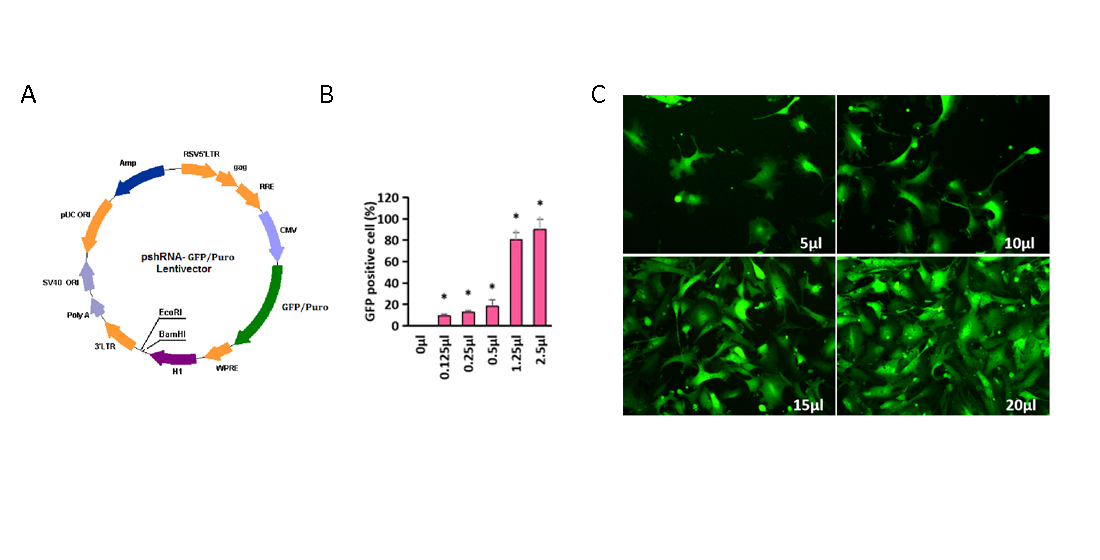

Supplement: Supplementary file 1 [file biomolecules-14-00509-s001.zip › Figure S1. shH19 virus transfection efficiency..tif]

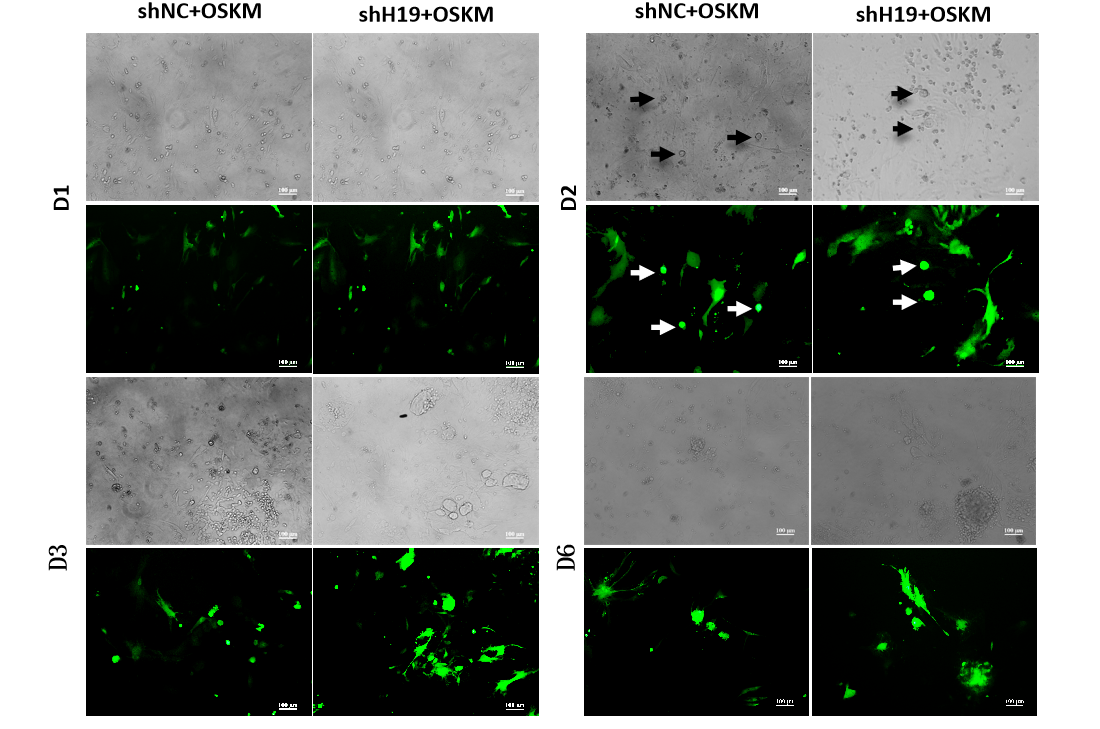

Supplement: Supplementary file 1 [file biomolecules-14-00509-s001.zip › Figure S2. The Morphological changes during ips reprogramming..tif]

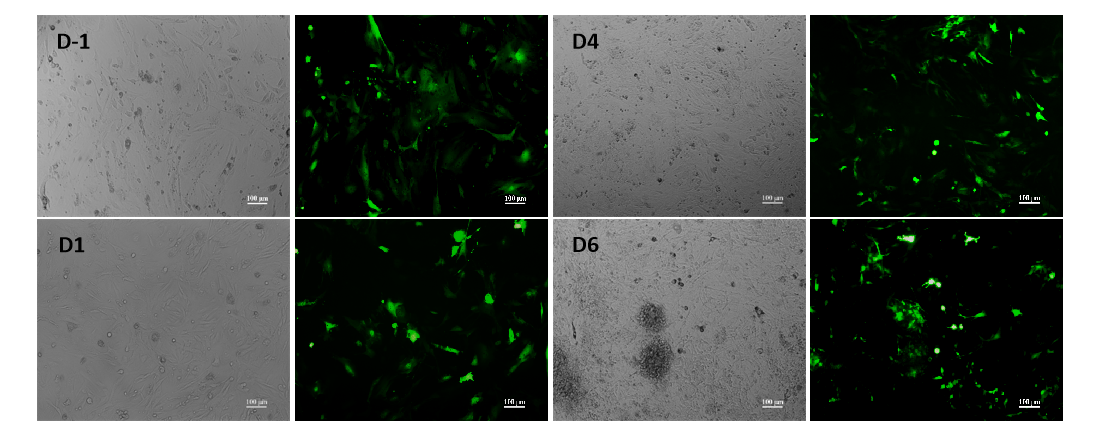

Supplement: Supplementary file 1 [file biomolecules-14-00509-s001.zip › Figure S3. The Morphology and GFP positive cells during shH19 virus infecting MEFs on different times..tif]

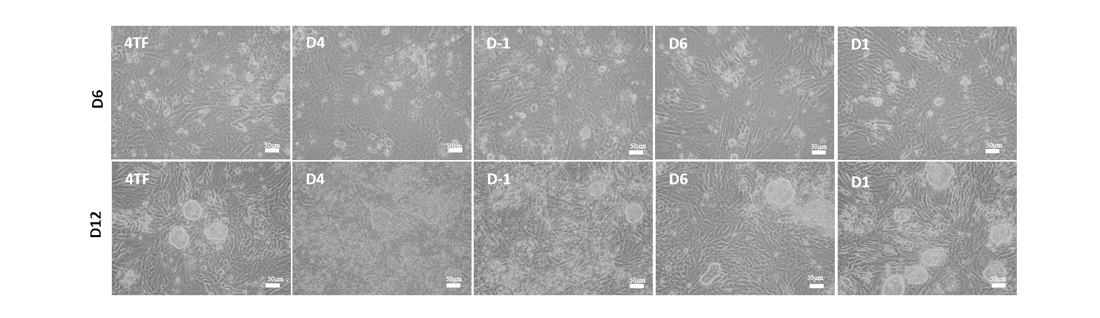

Supplement: Supplementary file 1 [file biomolecules-14-00509-s001.zip › Figure S4. The Morphology of ES clones on D6 and D12 during different groups..tif]

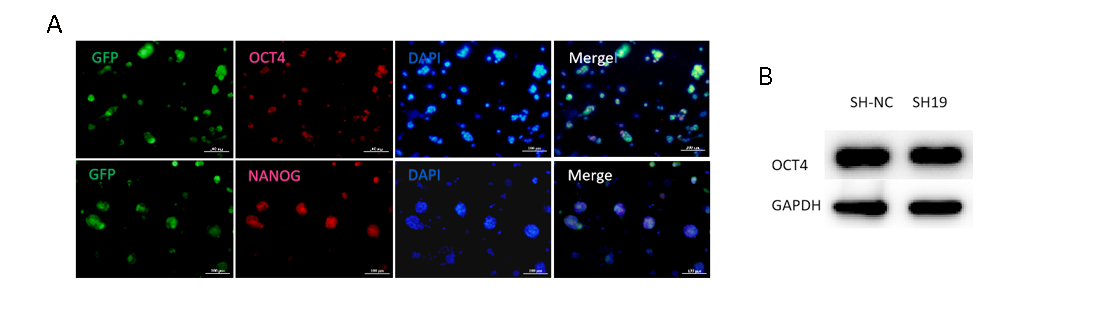

Supplement: Supplementary file 1 [file biomolecules-14-00509-s001.zip › Figure S5. shH19 iPSCs exhibits the expression of pluripotent genes, OCT4 and NANOG..tif]

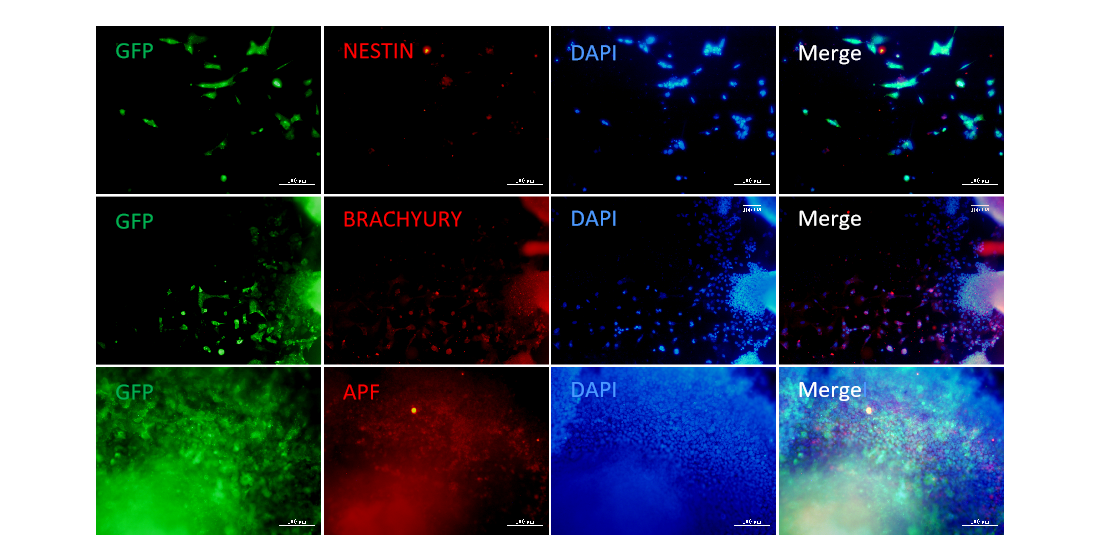

Supplement: Supplementary file 1 [file biomolecules-14-00509-s001.zip › Figure S6. EB of of SH-iPSCs lines obtained Germ layer genes, NESTIN, BRACHUYURY and AFP..tif]

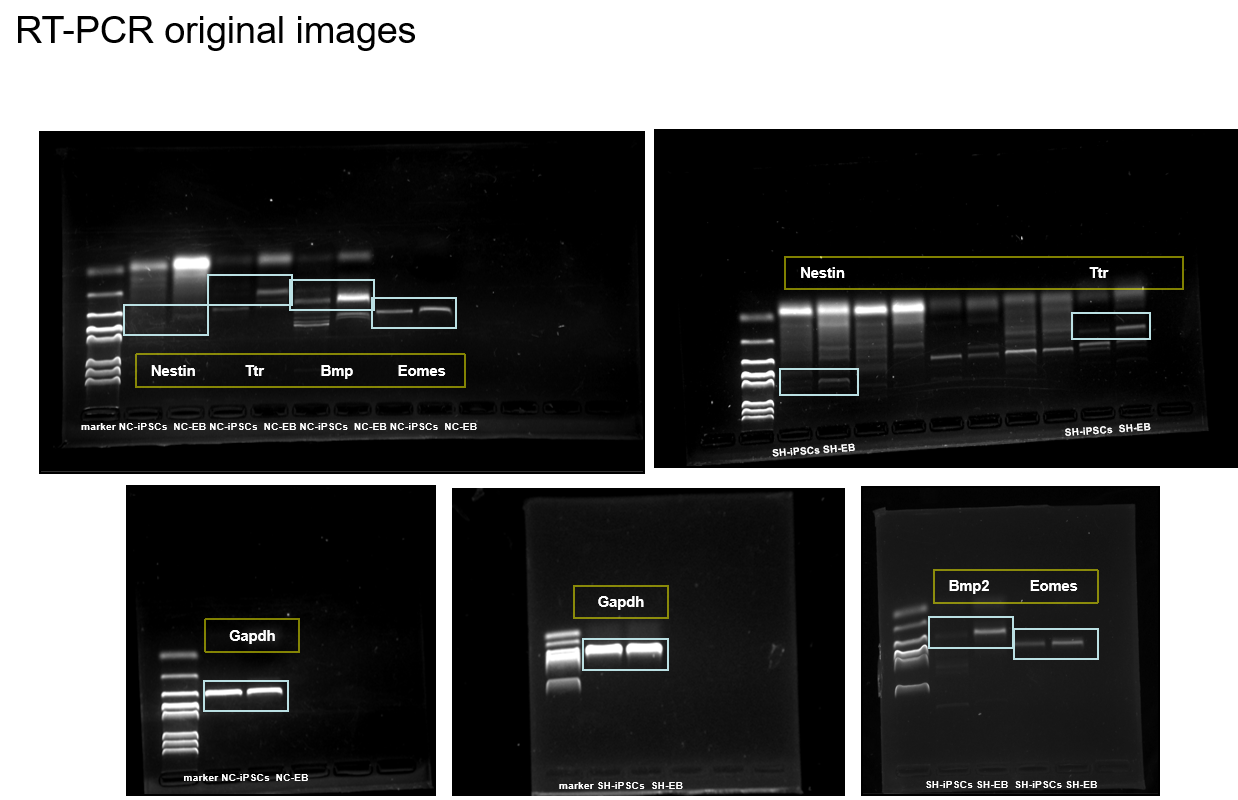

Supplement: Supplementary file 1 [file biomolecules-14-00509-s001.zip › RT-PCR original images.bmp]

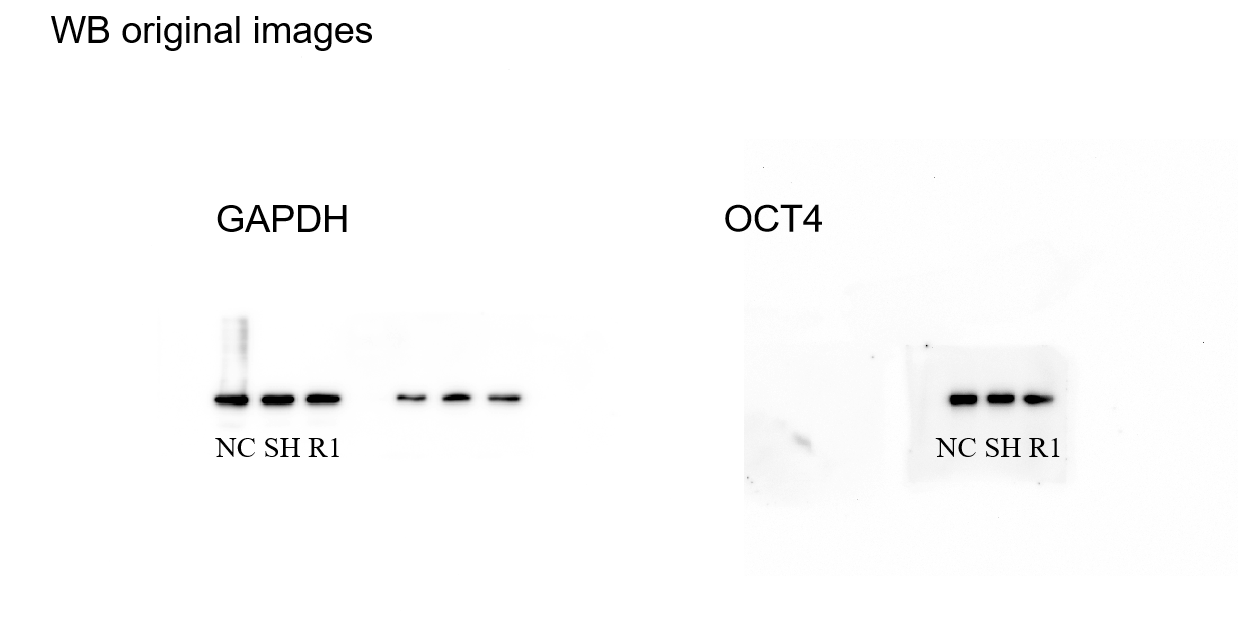

Supplement: Supplementary file 1 [file biomolecules-14-00509-s001.zip › WB-OCT4 and GAPDH.bmp]
